# Urinary-free cortisol-based thresholds for differentiating ACTH-dependent Cushing: a Spanish validation study

**DOI:** 10.1210/clinem/dgag187

**Published:** 2026-04-30

**Authors:** Betina Biagetti, Pedro Marques, Alfonso Soto-Moreno, Rogelio García-Centeno, Laura González-Fernández, María Dolores Ollero García, Ana Irigaray Echarri, Andres Cardona-Arias, María Dolores Moure Rodríguez, Miguel Paja, Ana Castro, Lucía Manzano Valero, Fernando Guerrero-Pérez, Cristina Lamas, Victoria Alcázar Lázaro, Paola Gracía, David Sanchis-Pascual, Cristina Álvarez-Escolá, Claudia Lozano-Aida, Felicia A Hanzu, Marta Araujo-Castro

**Affiliations:** Endocrinology and Nutrition Department, Department of Medicine, Hospital Universitario Vall d’Hebrón and Vall d′Hebron Research Institute (VHIR), Autonomous University of Barcelona, Reference Networks (ERN), Barcelona 08035, Spain; Pituitary Tumor Unit, Endocrinology Department, Hospital CUF Descobertas, Lisbon 1998-018, Portugal; Faculdade de Medicina, Universidade Católica Portuguesa, Lisbon 1649-023, Portugal; Endocrinology and Nutrition Department, Hospital Universitario Virgen del Rocío, Sevilla 41013, Spain; Endocrinology and Nutrition Department, Hospital Universitario Gregorio Marañón, Madrid 28007, Spain; Endocrinology and Nutrition Department, Hospital Universitario Gregorio Marañón, Madrid 28007, Spain; Endocrinology and Nutrition Department, Hospital Universitario Navarra, Pamplona 31008, Spain; Endocrinology and Nutrition Department, Hospital Universitario Navarra, Pamplona 31008, Spain; Endocrinology and Nutrition Department, Department of Medicine, Hospital Universitario Vall d’Hebrón and Vall d′Hebron Research Institute (VHIR), Autonomous University of Barcelona, Reference Networks (ERN), Barcelona 08035, Spain; Endocrinology and Nutrition Department, Cruces University Hospital, Biobizkaia Health Research Institute (IIS Biobizkaia), University of the Basque Country UPV/EHU, Bilbao 48903, Spain; Endocrinology and Nutrition, Biobizkaia Health Research Institute, Osakidetza, Basurto University Hospital, EHU, Basque Country University, Bilbao 48013, Bizkaia, Spain; Endocrinology and Nutrition Department, Hospital Universitario Toledo, Toledo 45007, Spain; Endocrinology and Nutrition Department, Hospital Universitario Toledo, Toledo 45007, Spain; Endocrinology and Nutrition Department, Hospital Universitario de Bellvitge, Cataluña ‘Hospitalet de Llobregat 08907, Spain; Endocrinology and Nutrition Department, Complejo Hospitalario Universitario de Albacete, Albacete 02006, Spain; Endocrinology and Nutrition Department, Hospital Universitario Severo Ochoa, Madrid 28914, Spain; Endocrinology and Nutrition Department, Hospital Universitario Royo Villanova, Zaragoza 50015, Spain; Endocrinology and Nutrition Department, Hospital La Fe, Valencia 46026, Spain; Endocrinology Department, Hospital Universitario La Paz, Madrid 28046, Spain; Endocrinology and Nutrition Department, Hospital Universitario Central de Asturias, Asturias 33011, Spain; Instituto de Investigación Sanitaria del Principado de Asturias (ISPA), Asturias 33011, Spain; Endocrinology and Nutrition Department, Hospital Clinic de Barcelona, FCRB-IDIBAPS, CIBERDEM, Barcelona 08036, Spain; Endocrinology and Nutrition Department, Hospital Universitario Ramón y Cajal, Madrid 28034, Spain; Instituto de Investigación Biomédica Ramón y Cajal (IRYCIS), Madrid 28034, Spain

**Keywords:** urinary-free cortisol, hypercortisolism, Cushing syndrome, Cushing disease, ectopic Cushing

## Abstract

**Context:**

Differentiating ectopic ACTH secretion (EAS) from Cushing disease (CD) remains one of the most challenging steps in the diagnostic workup of ACTH-dependent Cushing syndrome (CS). Urinary-free cortisol (UFC) expressed as times above the upper limit of normal (ULN) has been proposed as a simple, noninvasive discriminator, but external validation in independent populations is lacking.

**Objective:**

To validate the diagnostic performance of UFC × ULN for distinguishing EAS from CD and explore complementary biochemical markers, including late-night salivary cortisol (LNSC × ULN) and hypokalemia.

**Design, Setting, and Participants:**

Multicenter retrospective study from the Spanish Cushing Registry including 269 patients with ACTH-dependent Cushing's syndrome (208 CD, 61 EAS) diagnosed and managed in tertiary referral centers.

**Main Outcome Measures:**

Diagnostic accuracy of UFC × ULN and LNSC × ULN for discriminating EAS from CD, expressed as area under the ROC curve (AUC), sensitivity, specificity, and predictive value.

**Results:**

EAS patients were older (median 59.0 vs 44.9 years; *P* < .001) and showed higher UFC × ULN (16.6 vs 3.6; *P* < .001) and LNSC × ULN (9.3 vs 1.5; *P* < .001). UFC × ULN and LNSC × ULN achieved excellent discriminative performance (AUC 0.90 and 0.92). No EAS occurred with UFC × ULN < 3 × ULN, while 40.5% of patients with UFC ≥ 10 × ULN had EAS. The combination of severe hypercortisolism (UFC ≥ 10 × ULN and LNSC ≥ 9 × ULN) plus hypokalemia identified 75% of EAS with 98% specificity.

**Conclusion:**

UFC × ULN thresholds reliably stratify the probability of EAS vs CD. Severe hypercortisolism and hypokalemia strongly predict EAS, supporting a pragmatic diagnostic approach that prioritizes whole-body imaging in high-risk patients and pituitary-centered evaluation in mild cases.

Differentiating between Cushing disease (CD) and ectopic adrenocorticotropic hormone (ACTH) secretion (EAS) remains one of the most challenging aspects of the diagnostic workup for ACTH-dependent Cushing syndrome (CS) (1-3). Current guidelines (4) recommend pituitary magnetic resonance imaging (MRI) as the initial investigation, followed by bilateral inferior petrosal sinus sampling (BIPSS) or a noninvasive approach using a combination of 3 or 4 tests, specifically CRH (currently not available) (5) or desmopressin stimulation, followed by whole-body computed tomography (CT) when the etiology remains uncertain. BIPSS remains the gold standard for confirming a pituitary source of ACTH excess, providing excellent sensitivity and specificity when performed in experienced centers. However, the procedure is invasive, not universally available, and may pose a considerable risk in severely hypercortisolemic or medically unstable patients, in whom hypercoagulability, infection, and cardiovascular instability are frequent complications (6).

In clinical practice, the need for urgent therapeutic decisions in patients with severe CS often precludes the use of dynamic testing or invasive procedures (6, 7). Prompt identification of patients with a high probability of EAS is therefore critical to guide early imaging and therapeutic interventions while avoiding diagnostic delays related to complex localization studies.

In this context, the recent work by Lavoillotte et al. (8) proposed a pragmatic and noninvasive diagnostic approach based on the magnitude of 24 hours urinary-free cortisol (UFC) elevation. In a large bicentric cohort, UFC expressed as a times above the upper limit of normal (ULN) demonstrated excellent accuracy, with an area under the ROC curve (AUC) of 0.96 for distinguishing EAS from CD, outperforming plasma ACTH and other biochemical tests, including the desmopressin stimulation test. Stratification by UFC identified 3 clinically meaningful risk categories: <3 × ULN (0% EAS), 3-10 × ULN (6% EAS), and >10 × ULN (67% EAS). However, subsequent commentary has raised concerns about the applicability of this approach in patients with milder hypercortisolism, where small bronchial carcinoids may present with UFC values below <3 × ULN and coexist with incidental pituitary microadenomas (9). This underlines the need for independent, real-world validation of the proposed algorithm across broader clinical contexts.

The present study aimed to externally validate the diagnostic performance of UFC × ULN and explore whether integrating other biochemical variables could further enhance the sensitivity and specificity of this algorithm for the discrimination of ectopic vs pituitary ACTH-dependent CS.

## Patients and methods

### Study design and setting

This was a multicenter, retrospective observational study based on data from the SPAIN-CUSHING registry, a national database developed and coordinated by the Neuroendocrinology Area of the Spanish Society of Endocrinology and Nutrition (SEEN). The registry compiles real-world clinical, biochemical, and radiological information from patients with endogenous CS across multiple tertiary referral centers in Spain (10, 11). Data are retrospectively and prospectively entered into a standardized electronic case report form hosted in the REDCap SEEN platform.

The present analysis was conducted in November 2025. At that time, the registry included 429 patients with confirmed endogenous CS with available UFC data. Patients were diagnosed between [NOV 1985] and [JAN 2025]. The protocol was approved by the Ethics Committee of the Hospital Universitario Ramón y Cajal, Madrid, Spain (approval date: 26 November 2024, code: ACTA 472) and in each collaborating center. Patient consent was waived for retrospective cases without active follow-up and was requested only for patients who continued follow-up or who were prospectively included. The study followed the Strobe statement (12) and was conducted according to the mandates of the Declaration of Helsinki and good clinical practice guidelines.

### Study population

**Figure 1:**
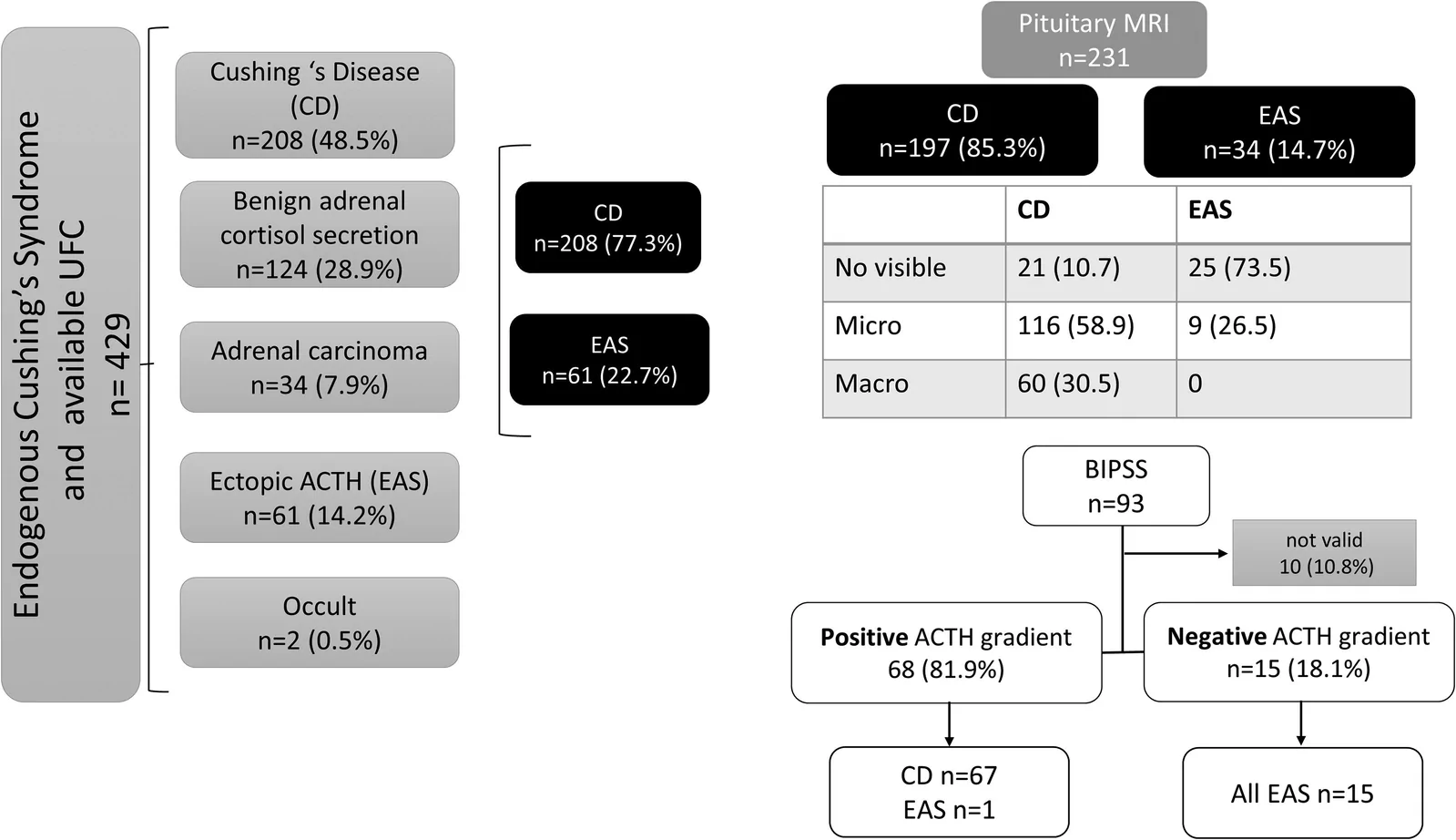
Patient selection and classification according to final etiology of ACTH-dependent CS. Flowchart summarizing the diagnostic distribution of 429 patients with endogenous Cushing syndrome (CS). Among 269 with ACTH-dependent Cushing syndrome, 208 (77.3%) had Cushing disease (CD) and 61 (22.7%) ectopic ACTH secretion (EAS). Pituitary MRI was performed in 231 patients, with an incidental lesion found in 9/34 (26.5%) EAS. BIPSS was available in 93 patients, of which 10 were technically invalid (9 in CD and 1 EAS). Among valid studies, a positive central-to-peripheral ACTH gradient was observed in 68 cases (including one false-positive), whereas all 15 negative gradients finally corresponded to ectopic ACTH secretion. Abbreviations: ACTH, adrenocorticotropic hormone; BIPSS, bilateral inferior petrosal sinus sampling; CD, Cushing Disease; EAS, ectopic ACTH secretion, UFC × ULN, urinary-free cortisol expressed as times above the upper limit of normal.

Among the 429 patients with endogenous CS and available UFC, those with ACTH-dependent CS were selected for this analysis. Cases of cortisol-secreting adrenal adenomas, adrenal hyperplasia, adrenocortical carcinoma and 2 cases of ACTH secretion of unknown origin, were excluded. The final study population comprised 269 patients, including 208 with CD and 61 with EAS, confirmed by histopathology, whole-body CT, or BIPSS when available. A detailed flowchart describing patient selection and diagnostic classification is provided in Fig. 1.

### Diagnostic definitions

The diagnosis and classification of CS followed the international guidelines in force at the time of diagnosis (4, 13, 14), integrating biochemical evidence of ACTH-dependent hypercortisolism, imaging findings, and, when available, invasive and histopathological confirmation. Importantly, registry inclusion did not require any predefined UFC threshold or specific hormonal cutoff.

In patients with CD with no visible pituitary lesion or with pituitary lesions <6 mm on MRI, a central-to-peripheral ACTH gradient on BIPSS was generally required, unless documented histopathological confirmation or postoperative biochemical remission. In operated patients, diagnostic confirmation was established by biochemical remission following transsphenoidal surgery, defined as a postoperative serum cortisol concentration <55 nmol/L (<2 µg/dL) or documented adrenal insufficiency consistent with remission (4). In cases where tissue samples were available, histological confirmation of an ACTH-secreting pituitary adenoma was obtained. Detailed subclassification according to WHO 2022 criteria (densely granulated, sparsely granulated, or Crooke cell subtypes) was not systematically available across centers (15, 16).

EAS was diagnosed based on histological confirmation of an extrapituitary ACTH-secreting neuroendocrine tumor (NET) including immunohistochemical staining for ACTH (17) and in some cases by the absence of an ACTH gradient on BIPSS combined with compatible imaging findings. In resected cases, postoperative biochemical remission or marked improvement of hypercortisolism was used as additional confirmation of tumor functionality.

### Hormonal assays and biochemical variables/indices

For all patients, UFC and late-night salivary cortisol (LNSC) were measured using immunoassay-based methods. Given the multicenter nature of the registry, results were standardized by expressing values relative to the ULN to reduce inter-assay variability. Plasma ACTH values were recorded in the morning under stable hypercortisolemic conditions, prior to the initiation of medical therapy. For patients with multiple baseline UFC, LNSC, and ACTH measurements obtained prior to treatment initiation, the value used for analysis was calculated as the mean of the two highest measurements. When only a single baseline measurement was available, that value was used.

Some biochemical indices were evaluated to assess the degree of hypercortisolism and its diagnostic value for distinguishing CD from EAS:

-UFC × ULN was categorized into 3 groups following the approach proposed by Lavoillotte et al (8) and consistent with the percentile distribution observed in our cohort:
- Group 1: <3 × ULN, compatible with mild or moderate hypercortisolism;
- Group 2: 3-9.9 × ULN, representing intermediate cortisol elevations; and
- Group 3: >10 × ULN, indicating severe hypercortisolism.
-LNSC x ULN was similarly stratified according to its percentile distribution and discriminative power in our cohort to reflect increasing cortisol burden:
- Group 1: 1.5 × ULN, mild elevations;
- Group 2: 1.5-8.9 × ULN, intermediate range; and
- Group 3: ≥9 × ULN, representing the highest percentile range and severe hypercortisolism.
- 8 mg Dexamethasone Suppression Test Index (8 mg DST-I, %): Percentage decrease in serum cortisol from baseline following a single 8 mg dexamethasone dose at 23:00 hours: 8 mg DST-I (%) = [1 − (post-dexamethasone cortisol/baseline cortisol)] × 100
- ACTH % change after desmopressin was defined as the percentage increase in plasma ACTH from baseline to peak following desmopressin stimulation.

All of these parameters were analyzed as continuous and categorical variables to evaluate their diagnostic performance in differentiating CD from EAS using receiver operating characteristic (ROC) analysis.

BIPSS was performed according to standard procedures for differential diagnosis of ACTH-dependent CS (2). A central-to-peripheral ACTH ratio ≥2 at baseline or ≥3 after stimulation was considered a positive gradient and indicative of a pituitary source, whereas ratios below these thresholds were interpreted as negative and suggestive of ectopic ACTH secretion.

Hypokalemia was defined as serum potassium below the lower limit of normal according to local laboratory reference ranges, either at diagnosis or in patients receiving potassium supplementation prior to its initiation.

### Statistical analysis

Continuous variables are expressed as median and range (min–max). Categorical variables are presented as counts and percentages. Group comparisons were performed using the Mann–Whitney *U* or Student *t* test for continuous variables, and the χ^2^ or Fisher exact test for categorical variables.

Diagnostic performance was evaluated using ROC curve analysis, with estimation of the AUC and 95% confidence intervals (CI). Comparisons between AUCs were performed using the DeLong test. Sensitivity, specificity, positive predictive value, and negative predictive value were calculated for pre-specified cut-offs and for the optimal threshold derived from the Youden index. All analyses were performed using Stata 16.0 (StataCorp, College Station, TX, USA). Two-tailed *P* values <0.05 were considered statistically significant.

## Results

### Patient characteristics

Overall, 269 patients had ACTH-dependent CS. According to final etiology, 208 (77.3%) had CD, and 61 (22.7%) had EAS (Fig. 1). Median follow-up differed significantly between groups, being longer in patients with CD than in those with EAS (9.3 [range 1.4-47.7] vs 4.9 [0.43-14.2] years; *P* < .001). The main clinical and biochemical characteristics of patients with CD and EAS are summarized in Table 1.

**Table 1:** Clinical and biochemical characteristics of patients according to ACTH-dependent Cushing syndrome subtype

Variable	n	Cushing disease (*n* = 208)	Ectopic ACTH syndrome (*n* = 61)	*P* value
**Age (years)**	269	44.9 (23.2-77.3)	59.0 (27.2-82.1)	<.001
**Female (%)**	269	157 (75.5)	30 (49.2)	<.001
**Baseline cortisol**	269	21.9 (12.1-72.4)	55.7 (15.2-153.0)	<.001
**Cortisol after 1 mg DST**	269	15.2 (2.3-25.2)	48.4 (14.1-149.2)	<.001
**UFC (× ULN)**	269	3.6 (1.2-77.2)	16.6 (4.9-329.1)	<.001
**Midnight salivary cortisol**	123	*n* = 98 1.5 (1.0-43.0)	*n* = 25 9.3 (1.6-139.2)	<.001
**Plasma ACTH (pg/mL)**	269	59.8 (22.0-120.2)	156.4 (42.0-5886.0)	<.001
**ACTH % change after desmopressin**	20	*n* = 18 43.8 (20.4-172.0)	*n* = 2 38.2 (−7.9-106)	.999
**8 mg DST-I (%)**	56	*n* = 49 75.0 (38.2-104.7)	*n* = 7 21.4 (16.1-41.3)	<.001
**Hypokalemia (%)**	261	*n* = 200 10 (5.0)	*n* = 61 51 (83.6)	<.001
**Pituitary MRI (%)**	231	*n* = 197	*n* = 34	<.001
**No visible lesion**	46	21 (10.7)	25 (73.5)	<.001
**<1 cm**	125	116 (58.9)	9 (26.5)	
**≥1c m**	60	60 (30.5)	0	
**Lesion size (mm)**	231	7.0 (1-42)	4.7 (2-7)	
**BIPSS**	93	*n* = 68	*n* = 15	<.001
**Not valid**	10	9 (11.8)	1 (5.9)	
**Pituitary source**	68	67 (88.2)	1 (5.9)	
**Ectopic source**	15	0	15 (88.2)	
**Metastatic**	265	0	38 (64.4)	<.001

Abbreviations: ACTH, adrenocorticotropic hormone; BIPSS, bilateral inferior petrosal sinus sampling; CD, Cushing disease; DST, dexamethasone suppression test; EAS, ectopic ACTH syndrome; MRI, magnetic resonance imaging; ULN, upper limit of normal; UFC, urinary-free cortisol.

Patients with EAS were older than those with CD (median: 59.0 vs 44.9 years; *P* < .001) and less frequently female (49.2% vs 75.5%; *P* < .001). All cortisol-related parameters were significantly higher in EAS compared with CD. Median LNSC was (9.3 vs 1.5), UFC × ULN was (16.6 vs 3.6 × ULN), and plasma ACTH levels (156.4 vs 59.8 pg/mL). Hypokalemia was present in 83.6% of EAS compared with 5.0% of CD (*P* < .001). Fifty-six patients underwent an 8 mg dexamethasone suppression test, with CD patients displaying a higher median cortisol suppression index in comparison with EAS patients (75.0% vs 21.4%, *P* < .001). The median ACTH percentage change after desmopressin administration was 43.8% (range 20.4-172.0) in 18 patients with CD and 38.2% (range 7.9-106.0) in 2 patients with EAS, with no statistically significant difference between groups (Table 1).

Pituitary MRI data were available in 231 patients. Of the 197 CD patients with MRI data available, 176 patients (89.3%) had a visible pituitary lesion, including 58.9% with lesions <1 cm and 30.5% with lesions ≥1 cm. In contrast, a pituitary lesion < 1 cm was detected in 26.5% of EAS cases, while 73.5% had no visible lesion on MRI. Among patients with a visible pituitary lesion, the median maximum lesion diameter was 7.0 mm (range 1-42) in CD and 4.7 mm (range 2-7) in EAS (*P* < .001) (Table 1).

**Figure 2:**
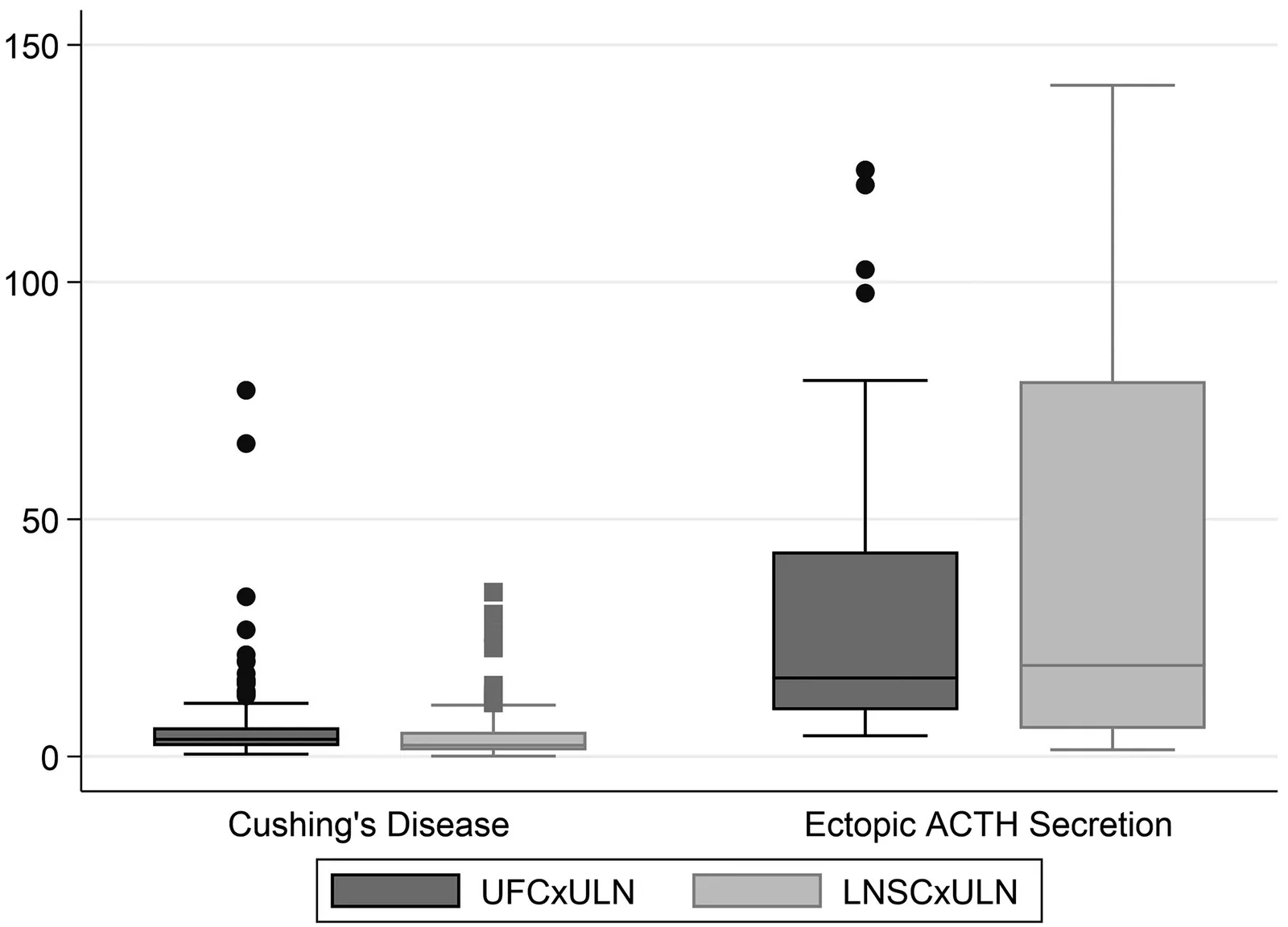
Comparison of cortisol burden (UFC × ULN and LNSC × ULN) across Cushing disease and ectopic ACTH secretion. Boxplots display individual and median values for UFC × ULN and LNSC × ULN in Cushing disease and ectopic ACTH secretion. In each box plot, the central horizontal line represents the median, and the box indicates the interquartile range (IQR; 25th–75th percentiles). Whiskers extend to the most extreme values within 1.5 × IQR from the box. Values beyond this range are plotted as individual points and considered outliers. (1 outliner with UFCxULN above 150 was excluded to allow visualization).

Figure 2 illustrates the distribution of cortisol burden in patients with CD and EAS, for both UFC × ULN and LNSC × ULN. Median values for both indices were markedly higher in EAS compared with CD, with wider dispersion and more extreme outliers reflecting the more severe hypercortisolism typically observed in EAS.

BIPSS was performed in 93 patients. Information on the stimulation protocol was available in 73 cases. Of these, 63 (86.3%) were stimulated with CRH and 10 (13.7%) with desmopressin. Ten procedures (10.8%) were classified as invalid due to technical issues, including catheterization failure, sampling problems, or other procedural errors. Among the valid BIPSS procedures, a central-to-peripheral ACTH gradient was observed in 68 patients. Of these, 67 were ultimately diagnosed with CD, and one corresponded to EAS from an olfactory neuroblastoma, representing a false-positive result. Conversely, all patients with a negative ACTH gradient were confirmed to have EAS.

Of the 61 patients with EAS, all underwent cross-sectional non-pituitary imaging according to local clinical practice. Whole-body CT was performed in 54 patients (88.5%), while a targeted CT protocol was used in 7 patients (11.5%), including chest CT alone in 5 cases and combined chest–abdominal CT in 2 cases. Functional imaging was frequently employed for complementing work-up: somatostatin receptor–based imaging (octreotide scintigraphy or ^68-Ga-DOTATOC PET/CT) was performed in 51 patients (83.6%) and FDG-PET in 55 patients (90.2%). Metastatic disease was identified in 38 of 61 (62.3%) EAS patients and in none of the CD patients.

### Histopathological findings

Of the 208 patients with CD, 159 have undergone transsphenoidal surgery at the time of the data analysis, and histopathological data reports were available for 127 patients, all of whom presented with ACTH-positive corticotroph tumors. In a subgroup of 54 patients, transcription factors were reported, with all tumors expressing T-PIT, with the majority being densely granulated corticotroph tumors (71.7%), followed by sparsely granulated corticotroph tumors (14.8%), Crooke cell tumors (11.1%), and other rare subtypes (2.4%).

Among the 61 patients with EAS, the diagnosis was confirmed in all cases by histological or cytological evidence of an EAS, obtained either from surgical specimens of the primary tumor or from biopsy of metastatic or extrapituitary lesions. Of these, 44 (72.1%) underwent surgical resection of the primary tumor or, when the primary remained occult, of a metastatic lesion, allowing full histopathological characterization of the primary tumor (Table 2). The remaining 17 (27.9%) did not undergo any surgical resection because of advanced metastatic disease or clinical contraindications.

**Table 2:** Histopathological subtypes of ectopic ACTH-producing tumors (*n* = 44)

Tumor category	Tumor subtypes	Total *N* = 44
**Well-differentiated NETs (G1–G2)**	Typical bronchial carcinoid, atypical bronchial/thymic carcinoid, thymic NET G2 (Ki-67 15-19%), pancreatic NET G1	16 (36.4%)
**Low-grade/NET not otherwise specified (NOS)**	Low-grade or NET NOS, probable pulmonary origin	4 (9.0%)
**Poorly differentiated NECs (G3)**	Small-cell lung carcinoma (SCLC/SCNEC), large-cell NEC (LCNEC), NEC G3 NOS, metastatic NEC	14 (31.8%)
**Rare non-pulmonary sources**	Medullary thyroid carcinoma, parathyroid carcinoma, olfactory neuroblastoma, adrenocortical–pituitary hybrid tumor	5 (11.4%)
**Only one metastatic lesion**	Liver, adrenal with unlocalized primary neuroendocrine neoplasm	5 (11.4%)

Abbreviations: LCNEC, large-cell neuroendocrine carcinoma; NEC, neuroendocrine carcinoma; NET, neuroendocrine tumor; NOS, not otherwise specified; SCLC, small-cell lung carcinoma; SCNEC, small-cell neuroendocrine carcinoma.

According to the 2022 WHO classification, 16 tumors (36.4%) were well-differentiated NETs (G1–G2), whereas 14 cases (31.8%) corresponded to poorly differentiated neuroendocrine carcinomas (NECs) either small-cell or large-cell types. Four additional cases (9.1%) were classified as low-grade NETs or NETs not otherwise specified (NET-NOS), and 5 cases (11.4%) represented rare or non-pulmonary origins. These included medullary thyroid carcinoma, parathyroid carcinoma, olfactory neuroblastoma, and one adrenocortical–pituitary hybrid tumor defined as a rare extrapituitary composite tumor showing immunohistochemical features of both adrenocortical tissue and ACTH-secreting pituitary differentiation (18). The remaining 5 patients (11.4%) corresponded to cases with a single metastatic lesion and occult primary tumor.

Overall, thoracic NETs accounted for nearly 65% of all histologically confirmed EAS cases, while high-grade NECs represented approximately one-quarter (Table 2). When tumors were stratified according to biological aggressiveness, well-differentiated or low-grade NETs accounted for 20 of 44 cases (45.5%), whereas the remaining 54.5% corresponded to poorly differentiated neuroendocrine carcinomas or aggressive non-pulmonary tumors, which are typically associated with overt hypercortisolism.

### Stratification according to urinary-free cortisol and late-night salivary cortisol values

EAS secretion was not observed in any patient with mild hypercortisolism (UFC × ULN <3). However, the prevalence of EAS increased to 12.5% among the ACTH-dependent CS patients with moderate hypercortisolism (3-9.9 × ULN) and 40.5% in the severe hypercortisolism group (≥10 × ULN) (*P* < .001).

As for LNSC × ULN, EAS accounted for 1 patient (3.5%) in the mild group (<1.5 × ULN), 7 patients (13.2%) in the moderate group (1.5-8.9 × ULN), and 16 patients (57.1%) in the severe group (≥9 × ULN). Both indices exhibited a clear dose–response relationship between cortisol burden and the likelihood of EAS.

**Figure 3:**
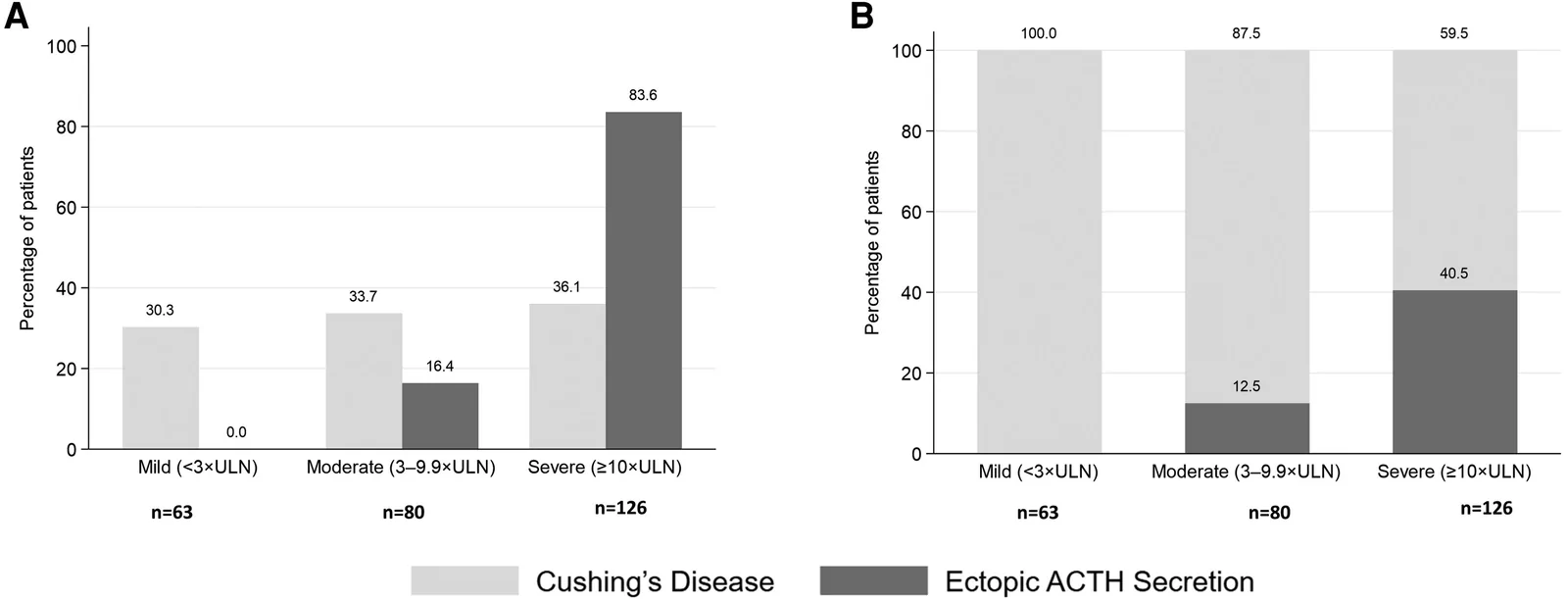
Severity of hypercortisolism in patients with Cushing disease and ectopic ACTH secretion according to UFC categories. Bar graph representation of the severity of hypercortisolism in patients with Cushing disease (light gray) and ectopic ACTH secretion (dark gray) across UFC × ULN categories (A) expressed as percentages within each etiology. Cushing disease shows a relatively even distribution across severity levels, whereas ectopic ACTH secretion is predominantly concentrated in the severe category. (B) Proportion of Cushing disease and ectopic ACTH secretion within each UFC × ULN severity group. Each bar represents 100% of patients within the corresponding severity category, illustrating the increasing probability of ectopic ACTH secretion with greater cortisol burden. Numbers below each category indicate the total number of patients in that UFC × ULN group.

As illustrated in Fig. 3A, while CD patients were relatively evenly distributed across mild (30.3%), moderate (33.7%), and severe (36.1%) categories, EAS patients were almost exclusively concentrated in the severe range (83.6%), with none identified in the mild category (0.0%). Figure 3B demonstrates that the probability of EAS within each UFC category increased progressively with higher cortisol burden, reaching 40.5% in the ≥10 × ULN group, while CD predominated in the lower categories.

**Figure 4:**
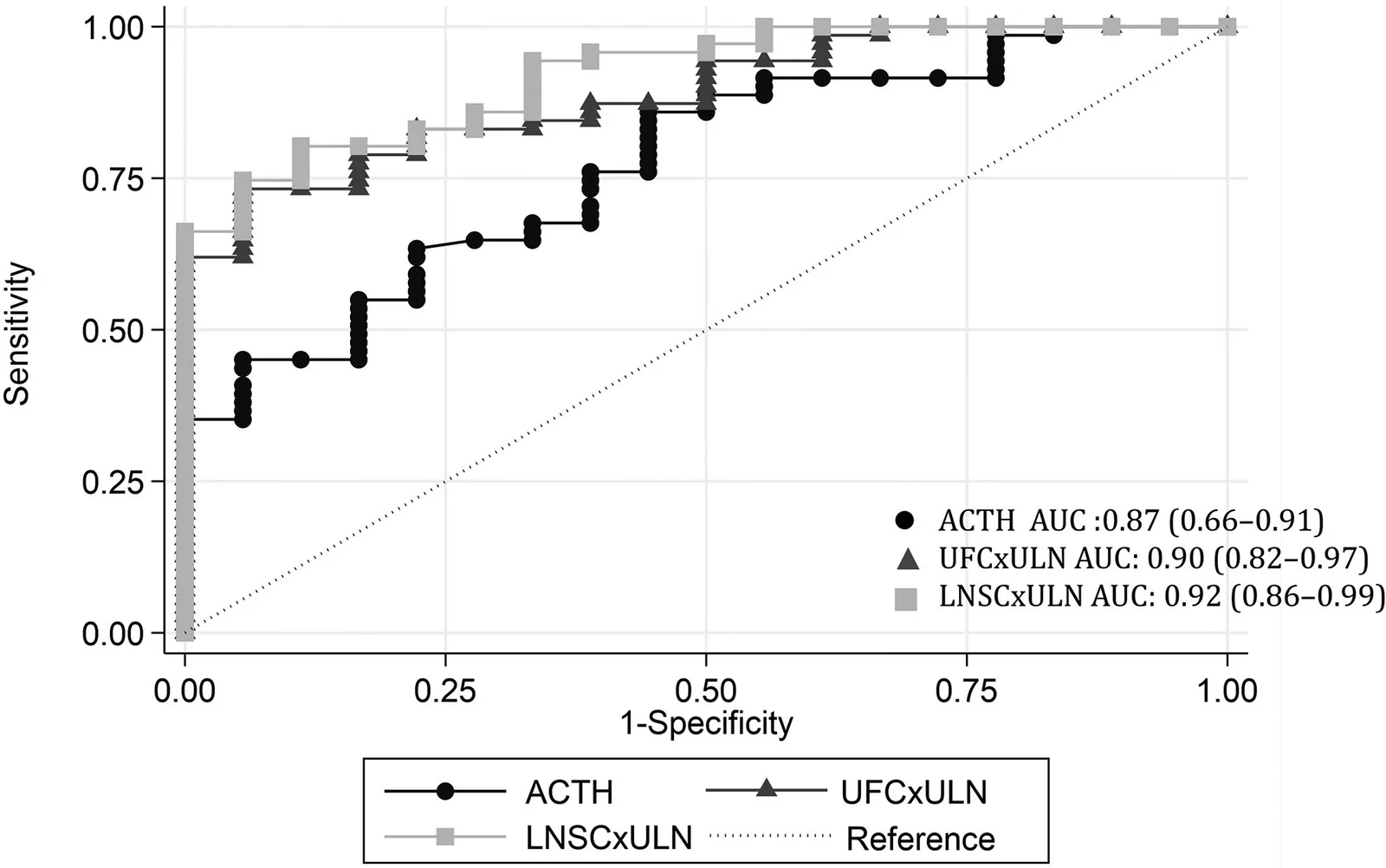
Receiver operating curves of biochemical parameters for differentiating ectopic ACTH secretion from Cushing disease. Receiver operating curves for differentiating ectopic ACTH secretion (EAS) from Cushing disease (CD) using plasma ACTH, 24-hour urinary-free cortisol expressed as times above the upper limit of normal (UFC × ULN), and late-night salivary cortisol (LNSC × ULN). LNSC × ULN (AUC = 0.92; 95% CI 0.86-0.99) and UFC × ULN (AUC = 0.90; 95% CI 0.82-0.97) demonstrated excellent discriminative performance, outperforming ACTH (AUC = 0.87; 95% CI 0.66-0.91). The overall comparison between curves was statistically significant (*P* = .022).

The diagnostic performance of biochemical parameters for differentiating EAS from CD is shown in Table 3. From all parameters evaluated, LNSC × ULN and UFC × ULN achieved the highest discriminative power, with AUCs of 0.92 (95% CI 0.86-0.98) and 0.89 (95% CI 0.82-0.96), respectively. Their ROC curves showed a clear superiority over baseline ACTH, which reached an AUC of 0.77 (95% CI 0.66-0.89) with a *P* value of 0.022 for the overall comparison (Fig. 4).

**Table 3:** Diagnostic performance of biochemical parameters for differentiating ectopic ACTH syndrome (EAS) from Cushing disease (CD)

Biochemical parameter	AUC (95% CI)	Optimal cutoff	Sensitivity (%)	Specificity (%)	Youden Index	PPV (%)	NPV (%)
**Baseline ACTH (pg/mL) *n* = 269**	0.87 (0.66-0.91)	≥ 120	73	88	0.61	71	87
**UFC** × **ULN *n* = 269**	0.90 (0.82-0.97)	≥ 10	76	88	0.64	80	85
**DST 1 mg (nmol/L) *n* = 242**	0.83 (0.75-0.91)	≥ 25	74	84	0.58	70	86
**LNSC** × **ULN *n* = 123**	0.92 (0.86-0.99)	≥ 9.1	83	82	0.65	77	87
**Cortisol suppression index *n* = 56**	0.69 (0.53-0.84)	≥ 70%	78	56	0.34	64	72

Abbreviations: ACTH, adrenocorticotropic hormone; AUC, area under the receiver operating characteristic curve; DST 1 mg, 1 mg overnight dexamethasone suppression test; PPV, positive predictive value; NPV, negative predictive value; ULN, upper limit of normal.

### Comparative prevalence of EAS and pituitary MRI findings according to the UFC × ULN categories

Table 4 summarizes the comparative distribution of EAS according to UFC × ULN categories across our cohort and in the series reported by Lavoillotte et al. and additionally details the prevalence of positive pituitary MRI findings in CD and EAS in our cohort. In both series, the prevalence of EAS increased markedly with higher cortisol burden, while no EAS cases were observed in patients with UFC < 3 × ULN (Table 4). In the present study, EAS prevalence rose from 12.5% in the intermediate UFC category (3-9.9 × ULN) to 40.5% in patients with UFC ≥10 × ULN.

**Table 4:** Comparative prevalence of ectopic ACTH secretion according to UFC × ULN categories

	Lavoillotte study	Present study				
UFC × ULN group	EAS prevalence (12.7%) *n* = 36/283	EAS prevalence (22.7%) *n* = 61/269	Pituitary MRI EAS *n* = 34		Pituitary CD *n* = 197	
			Positive *n* = 9 n (%)	Lesion ≥6 mm n (%)	Positive *n* = 176 n (%)	Lesion≥6 mm n (%)
< 3 × ULN	0% (0/126)	0% (0/63)	—	—	51/61 (83.6)	31/51 (60.8)
3-9.9 × ULN	6.1% (7/114)	12.5% (10/80)	4/8 (50.0)	1/4 (25.0)	57/65 (87.7)	37/57 (64.9)
≥ 10 × ULN	67.4% (29/43)	40.5% (51/126)	5/26 (19.2)	0	68/71 (95.8)	44/68 (64.7)

Abbreviations: ACTH, adrenocorticotropic hormone; CD, Cushing disease; EAS, ectopic ACTH syndrome; MRI, magnetic resonance imaging; ULN, upper limit of normal; UFC, urinary-free cortisol.

Importantly, among high-risk patients (UFC ≥10 × ULN), pituitary MRI identified incidental pituitary lesions in 5 of 26 patients with EAS (19.0%), all of which were small (<6 mm) and therefore non-diagnostic, still requiring BIPSS for source confirmation. In contrast, among patients with CD, pituitary MRI revealed lesions <6 mm in 24 of 68 cases (35.3%), again necessitating further testing to confirm pituitary origin (Table 4).

### Assessing combined biochemical parameters: UFC × ULN, LNSC × ULN, and hypokalemia

In our cohort, concordantly severely elevated cortisol indices (UFC ≥10 × ULN and LNSC ≥9.9 × ULN) were found in 37.9% of patients. This biochemical profile was highly characteristic of EAS, being present in 47 of 61 (77.1%) EAS cases compared to 55 of 208 (26.4%) CD patients (*P* < .001). When hypokalemia was added to UFC, the discriminative power further increased: 75.4% of EAS vs only 1.9% of CD met both criteria (UFC ≥10 × ULN + hypokalemia; *P* < .001).

## Discussion

This multicenter study provides external validation of the UFC-based diagnostic algorithm proposed by Lavoillotte et al (8) in a large independent Spanish cohort of 269 patients with ACTH-dependent CS. Our findings confirm that UFC × ULN has excellent discriminative performance in distinguishing EAS from CD, with a stratified risk approach identifying 3 distinct groups: no EAS risk (<3 × ULN), low-to-moderate risk (3-10 × ULN), and high risk (>10 × ULN). These results may support a paradigm shift towards a biochemistry-first approach rather than imaging-first in the differential diagnostic workup of ACTH-dependent CS, particularly in patients with severe hypercortisolism.

Our study validates the diagnostic accuracy of UFC × ULN in an independent population, where we obtained an AUC of 0.90 (95% CI 0.82-0.97), similar to the results of Lavoillotte et al. (8) (AUC of 0.96 (95% CI 0.93-0.99), notably for the overlapping CI and excellent discriminative performance. The stratification by UFC levels yielded remarkably similar prevalence patterns to the original study (8), with EAS becoming increasingly prevalent as cortisol burden rose. However, our series included a higher proportion of EAS cases (22.7% vs 12.7% in Lavoillotte main cohort). Notably, the proportion of EAS in the severe UFC × ULN group (≥ 10 × ULN) was lower in our study (40.5% vs 67.4%). This suggests that, even at very high UFC levels, a considerable subset of patients in real-world endocrine practice still harbor CD. Although the proportion of EAS among patients with UFC ≥10 × ULN was lower than that reported by Lavoillotte et al, both cohorts demonstrate the same consistent biological gradient: the probability of EAS increases sharply with rising cortisol burden. Differences in absolute proportions likely reflect referral patterns, cohort composition, and real-world heterogeneity rather than methodological discordance. Furthermore, biological heterogeneity within CD itself may contribute to this observation. Specifically, a subset of patients with CD may exhibit disproportionately severe hypercortisolism and atypical hormonal responses, such as glucocorticoid-induced positive feedback (GIPF), defined as a paradoxical increase in ACTH and/or cortisol secretion following glucocorticoid administration, attributed to altered glucocorticoid receptor signaling in corticotroph tumors (19). In a recent multicenter study from the same national registry, GIPF was identified in approximately 9% of patients with CD and was associated with higher UFC levels and poorer surgical outcomes (19). This phenotype may partly explain why a non-negligible proportion of patients with UFC ≥10 × ULN in real-world cohorts still have pituitary disease, emphasizing the need for clinical judgment before bypassing pituitary evaluation. Despite these population differences, the overall diagnostic trend remains robust: EAS prevalence increases with rising UFC × ULN, whereas CD predominates in the lower and intermediate ranges.

Lamas’ commentary raised legitimate concerns about the applicability of UFC-based stratification in patients with milder hypercortisolism, particularly those with small bronchial NETs that may present with UFC < 3 × ULN and coexist with incidental pituitary microadenomas (9). Our findings, consistent with Laviolette French–Belgian cohorts, showed no EAS cases with UFC <3 × ULN. However, this observation should be interpreted with caution, given the characteristics of our cohort. In fact, in our registry, the majority of EAS tumors were poorly differentiated NECs or aggressive non-pulmonary neoplasms, entities that typically present with marked cortisol excess, likely enriching the cohort for patients with more marked cortisol excess. As such, the lack of EAS cases with UFC <3 × ULN may reflect the clinical profile of the population included rather than a definitive diagnostic cutoff.

The absence of EAS cases with UFC <3 × ULN in our cohort and that of Lavoillotte et al (8) does not negate the existence of mild EAS, as appropriately highlighted by Lamas (9). Rather, it suggests that within referral populations with clinically overt ACTH-dependent CS, a UFC threshold <3 × ULN is associated with a low observed probability of EAS. In addition, in patients with mild hypercortisolism, UFC determination may be less frequently requested in routine practice and less frequently registered, potentially contributing to the underrepresentation of mild ectopic cases in registry-based analyses. Furthermore, small bronchial carcinoids may present with mild, intermittent, or cyclic hypercortisolism and can biochemically mimic pituitary disease (20-25); such presentations may be under-represented in endocrine registries because of referral patterns and a potential bias toward registry more severe cases.

Hypokalemia was markedly more frequent in EAS (83.6%) compared with CD (5.0%). This difference is most likely related to the greater magnitude of hypercortisolism in EAS, as reflected by significantly higher median UFC levels (16.6 × ULN vs 3.6 × ULN in CD, *P* < 0.001), rather than to a distinct underlying pathophysiological mechanism. In addition, a more abrupt or clinically overt presentation in some EAS cases may contribute to more pronounced electrolyte disturbances. Serum potassium should therefore be interpreted as a marker of overall cortisol burden rather than as an independent discriminator of etiology.

The key distinction therefore lies in the clinical presentation and diagnostic urgency. In patients with severe(UFC ≥10 × ULN) and consistent biochemical features, prioritizing whole-body imaging is both safe and time-efficient, as our data confirm a 40.5% EAS prevalence in this group. Conversely, in patients with mild hypercortisolism (UFC <3 × ULN), a pituitary-first approach with MRI and, when indicated, BIPSS remains essential to avoid misdiagnosing rare but documented cases of ectopic ACTH secretion that present with modest cortisol elevations (20-25).

On this basis, we propose a risk-stratified diagnostic approach that integrates UFC × ULN, hypokalemia, and clinical judgement. In patients with severe hypercortisolism (UFC ≥10 × ULN), particularly when accompanied by hypokalemia, early evaluation for an ectopic source using the most readily available cross-sectional imaging modality (typically whole-body CT) may be prioritized, especially in clinically unstable or severe cases. Functional imaging may be incorporated according to local availability and clinical suspicion. Importantly, this strategy is not intended to bypass pituitary MRI or BIPSS, which remain essential components of the diagnostic algorithm when imaging findings are inconclusive or discordant. In contrast, in moderate and mild presentations, evaluation should remain pituitary-focused, with MRI as the initial step, followed by BIPSS if the diagnosis remains uncertain, particularly when an incidental pituitary lesion coexists with atypical features or when the clinical presentation does not fully align with typical CD.

This stratified approach acknowledges that while the UFC-based algorithm proposed by Lavoillotte et al (8) is highly valuable for triaging patients with severe hypercortisolism, it should not replace comprehensive evaluation in patients with mild disease, where rare ectopic sources can be easily missed.

An additional observation from our study was the relatively high rate of pituitary MRI positivity in patients with CD (89.3%), exceeding the approximately 70 to 77% reported in prior series (26). This finding may reflect contemporary high-resolution imaging techniques as well as the diagnostic pathways typical of tertiary referral centers and registry-based cohorts. We also observed a high prevalence of pituitary incidentalomas in patients with EAS. Among the 34 EAS patients who underwent pituitary MRI, 9 (26.5%) harbored a pituitary lesion, a prevalence far exceeding that reported in the general population (27). This finding highlights the substantial risk of diagnostic misdirection when an incidental pituitary lesion coexists with EAS (particularly when the lesion is larger than 6 mm), as it may be erroneously interpreted as causative and lead to pituitary surgery without adequate confirmatory testing, including BIPSS. In addition, in patients with CD and UFC ≥10 × ULN, pituitary MRI revealed lesions <6 mm in up to 33% of cases, once again necessitating further testing. In this context, a UFC-based diagnostic algorithm may help reduce inappropriate pituitary surgery by prompting early whole-body imaging in patients with a clearly elevated cortisol burden (≥10 × ULN).

Nevertheless, caution remains warranted when applying a “CT-first” approach to patients with UFC ≥10 × ULN. Whole-body imaging frequently reveals incidental findings (such as benign lung nodules), potentially leading to diagnostic confusion or unnecessary interventions. In this context, suspicious findings on whole-body CT should be further evaluated with appropriate functional imaging studies such as DOTATOC-PET or FDG-PET (4, 6, 28). Importantly, in inconclusive cases, BIPSS remains the gold standard for confirming a pituitary source and should not be omitted solely on the basis of severe hypercortisolism.

A novel finding in our study was the excellent performance of LNSC × ULN (AUC 0.92, 95% CI 0.86-0.98), which numerically (though not significantly) outperformed UFC × ULN (AUC 0.89). LNSC × ULN stratification yielded a similarly progressive increase in EAS prevalence: 3.5% (<1.5 × ULN), 13.2% (1.5-8.9 × ULN), and 57.1% (≥9 × ULN). LNSC offers several practical advantages such as point-in-time sampling vs 24-hour UFC collection (better patient compliance, fewer collection errors) and is less affected by renal dysfunction or incomplete collections (4). However, LNSC standardization remains challenging due to assay variability and the lack of universal cutoffs. In clinical practice, and based on our findings in this study, we propose LNSC as (1) a complementary marker when UFC results are borderline or unreliable; (2) an alternative in patients unable to complete accurate 24-hour UFC collections; and as (3) a corroborative test, whereby concordantly elevated UFC and LNSC values strengthen confidence in the presence of severe hypercortisolism.

The combined assessment of cortisol burden and hypokalemia provided additional diagnostic discrimination in EAS. While severe hypercortisolism alone (UFC ≥10 × ULN and LNSC ≥9 × ULN) was strongly associated with EAS, the coexistence of hypokalemia markedly increased specificity. This combined biochemical profile was observed in 3 of every 4 patients with EAS, but in only 1.9% of those with CD.

Our study has limitations. The multicenter design involved diverse local protocols, heterogeneous biochemical assays for cortisol and ACTH (immunoassay-based, rather than mass spectrometry), and variable imaging quality and interpretation across institutions. These factors, along with incomplete test availability in some cases, may have introduced minor inter-center variability. Another limitation of our study is the relatively high proportion of patients with severe hypercortisolism, as assessed by UFC. This finding likely reflects a selection bias related to both the study design and the structure of the registry. First, inclusion in the present analysis required the availability of UFC measurements, a test that in routine clinical practice is more frequently requested in patients with clinically overt hypercortisolism, whereas patients with mild, borderline, or diagnostically uncertain presentations are often initially evaluated with simpler first-line tests such as the 1-mg DST or LNSC and may not undergo repeated or complete UFC assessment. Second, the Spanish Cushing Registry is not a population-based registry, and case reporting is not systematic nationwide. As a result, patients with mild or unresolved hypercortisolism may be underreported, while cases with confirmed diagnoses and more complete biochemical characterization are preferentially captured. Therefore, caution is warranted when extrapolating our findings to populations with predominantly mild or subclinical ACTH-dependent hypercortisolism, in whom the distribution of UFC levels and the relative prevalence of ectopic ACTH secretion may differ. Finally, serum dexamethasone concentrations to biochemically confirm adequate exposure during suppression testing were not measured. However, this reflects routine clinical practice in most participating centers, and dexamethasone suppression results were not central to the primary diagnostic stratification or predictive analyses of this study. Nevertheless, the consistency of trends across subgroups strengthens the validity of our findings.

In summary, our results support the use of a pragmatic diagnostic pathway: in patients with severe hypercortisolism and hypokalemia, proceeding directly to whole body imaging is reasonable and time-efficient, whereas in cases with mild cortisol elevations and normal potassium, a pituitary-first strategy with MRI and BIPSS remains essential. This two-tiered approach reconciles the strengths of both Lavoillotte and Lamas’ frameworks, combining biochemical precision with clinical judgment, and may improve both diagnostic accuracy and timeliness of management in ACTH-dependent CS.

## Data Availability

The authors agree to make data and materials supporting the results or analyses presented in their paper available upon reasonable request.
